# Report of DeSanto-Shinawi Syndrome in Three Boys With Two Novel Variants in the WAC Gene and Expansion of the Phenotype

**DOI:** 10.7759/cureus.70845

**Published:** 2024-10-04

**Authors:** Zuhair Rahbeeni, Nabilah Alwadani, Maryam Al-Shehhi, Eisa A Faqeih, Sarar Mohamed

**Affiliations:** 1 Genetics, King Faisal Specialist Hospital and Research Centre, Riyadh, SAU; 2 Genetics, Sheikh Khalifa Medical City, Abu Dhabi, ARE; 3 Genetics, King Fahad Medical City, Riyadh, SAU; 4 Genetics, Prince Sultan Military Medical City, Riyadh, SAU; 5 Collage of Medicine, Alfaisal University, Riyadh, SAU; 6 Faculty of Medicine, National University, Khartoum, SDN

**Keywords:** desanto-shinawi syndrome, expansion, novel, phenotype, variant, wac gene

## Abstract

Desanto-Shinawi syndrome (DESSHS) is a rare autosomal dominant disorder caused by a loss of function variant or deletion of the WAC gene. DESSHS is characterized by dysmorphic features and global developmental delay. In this report, we discuss three boys with DESSHS. These three patients exhibited the characteristic facial dysmorphism, intellectual disability, and behavioral problems associated with DESSHS. In addition, the patients presented with features not previously associated with DESSHS, including choanal atresia, flat feet, hypospadias, caudothalamic groove, and periventricular leukomalacia. Two of our patients had novel sequence variants (de-novo) in the WAC gene, specifically c.921del (p. E307Dfs*24) and c.479delC (p. Pro160fs*32). The third variant was a 9,389 kbp deletion on chromosome 10p12.31p11.22, resulting in the deletion of 74 genes, including the WAC gene. This report highlights manifestations not previously reported with DESSHS and may expand the understanding of this rare disorder. Furthermore, two new variants were detected in our patients.

## Introduction

Desanto-Shinawi syndrome (DESSHS, OMIM #616708) is an autosomal dominant disorder characterized by global developmental delay, behavioral abnormalities, and characteristic dysmorphic facial features [1]. DESSHS was described by DeSanto et al. in 2015 [1]. In the last decade, many cases have been reported from around the world including in China, Italy, South America, Portugal, Bahrain, Japan, Canada, and Turkey [1-9]. DESSHS is caused by loss of function variants or microdeletion of the WAC gene, WW domain-containing adaptor with coiled-coil, located on chromosome 10p12.1 [1]. This gene encodes for the WAC protein [10,11]. A recent translational study explored the structure of the WAC protein, its cellular localization, function, and evolution [10]. This protein contains amino acids and phosphorylation signals, which are likely to be involved in gene transcription, cellular signaling, and autophagy [6,10,11]. To elucidate the molecular mechanism and function of the WAC gene further, a vertebrate model has been developed including both murine and zebrafish [12]. These models develop similar phenotypes to DESSHS including craniofacial and behavioral abnormalities [12]. Furthermore, the mouse model shows susceptibility to developing seizures [12]. 

There were over 30 cases of DESSH described in the literature that were caused by point mutations [1-9]. On the other hand, deletion encompassing the WAC gene was reported in over 10 cases [1-9]. The phenotype of patients with DESSH seems to be influenced by the genotype to some extent [11]. Some features like malar flattening and prominent forehead tend to be associated with point mutation while facial dysmorphic features, specifically deep-set eyes, synophrys, and depressed nasal bridge seem to be more common in WAC deletion [11]. However, the phenotypic genotypic spectrum of DESSHS is yet to be fully defined. Here, we report three unrelated patients with some clinical manifestations not described before in association with DESSHS, two with novel sequence variants in the WAC gene, and a third one with a deletion encompassing the WAC gene.

## Case presentation

Table 1 shows the summarized developmental, neurological, dysmorphic, and other clinical presentations of the three patients with DESSH.

**Table 1:** Features of the three patients with Desanto-Shinawi syndrome

	Case 1	Case 2	Case 3
Gender	Male	Male	Male
Intellectual disability	Yes	Yes	Yes
Motor delay	Yes	Yes	Yes
Speech delay	Yes	Yes	Yes
Attention deficit	Yes	No	No
Hyperactivity	Yes	No	No
Aggression	Yes	No	No
Sleep disturbance	No	Yes	No
Hypotonia	Yes	Yes	No
Epilepsy	No	No	Yes
Dysmorphic feature	High forehead, midface retrusion, downslanting palpebral fissure, deep-set eyes, synophyrs, and low-set ears	Square-shaped face, midface hypoplasia with bilateral ptosis, deep-set eyes, flat nasal bridge, bulbous nasal tip, and low-set ears	large broad forehead, square-shaped face, almond eyes, depressed nasal bridge, thick lips, and low-set ears
Congenital heart disease	No	No	Yes (partial anomalous pulmonary venous return)
Feeding difficulties	No	Yes	Yes
Constipation	Yes	Yes	Yes
Other abnormalities		Eczema on hands and feet	Choanal atresia, hypospadias, undescended testis, hydrocephalus, ectopic left kidney

Case 1

**Figure 1:**
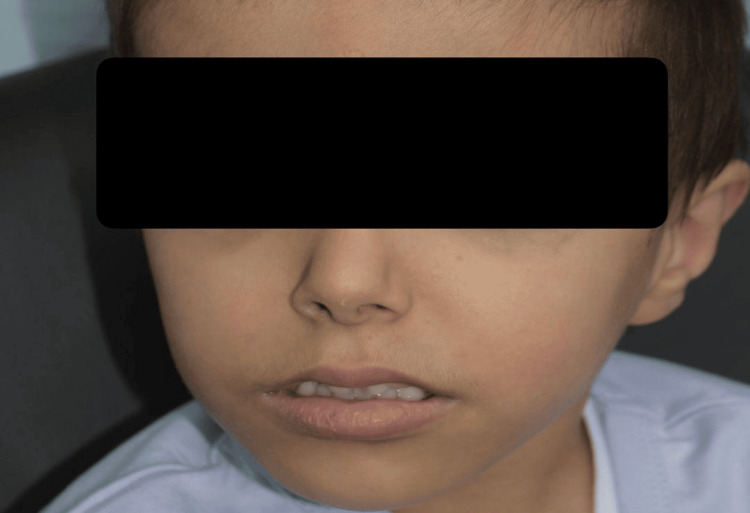
Eight-year-old Saudi boy with DeSanto-Shinawi Syndrome (case 1)

This is an eight-year-old Saudi boy born into a consanguineous marriage. He presented to the clinic in the first two years of life with intellectual, motor, and speech delays with attention deficit, hyperactivity disorder, aggression, and hypotonia (Table 1). Dysmorphic features included a high forehead, midface retrusion, downslanting palpebral fissure, deep-set eyes, synophyrs, and low-set ears (Figure 1). He had feeding difficulties and constipation. He had limited movement of the bilateral ankle joints since birth. Whole-exome sequencing showed a heterozygous likely pathogenic novel variant in the WAC gene, c.479 delC (p. Pro160fs*32). Carrier testing of this variant for his parents showed a wild type.

Case 2

**Figure 2:**
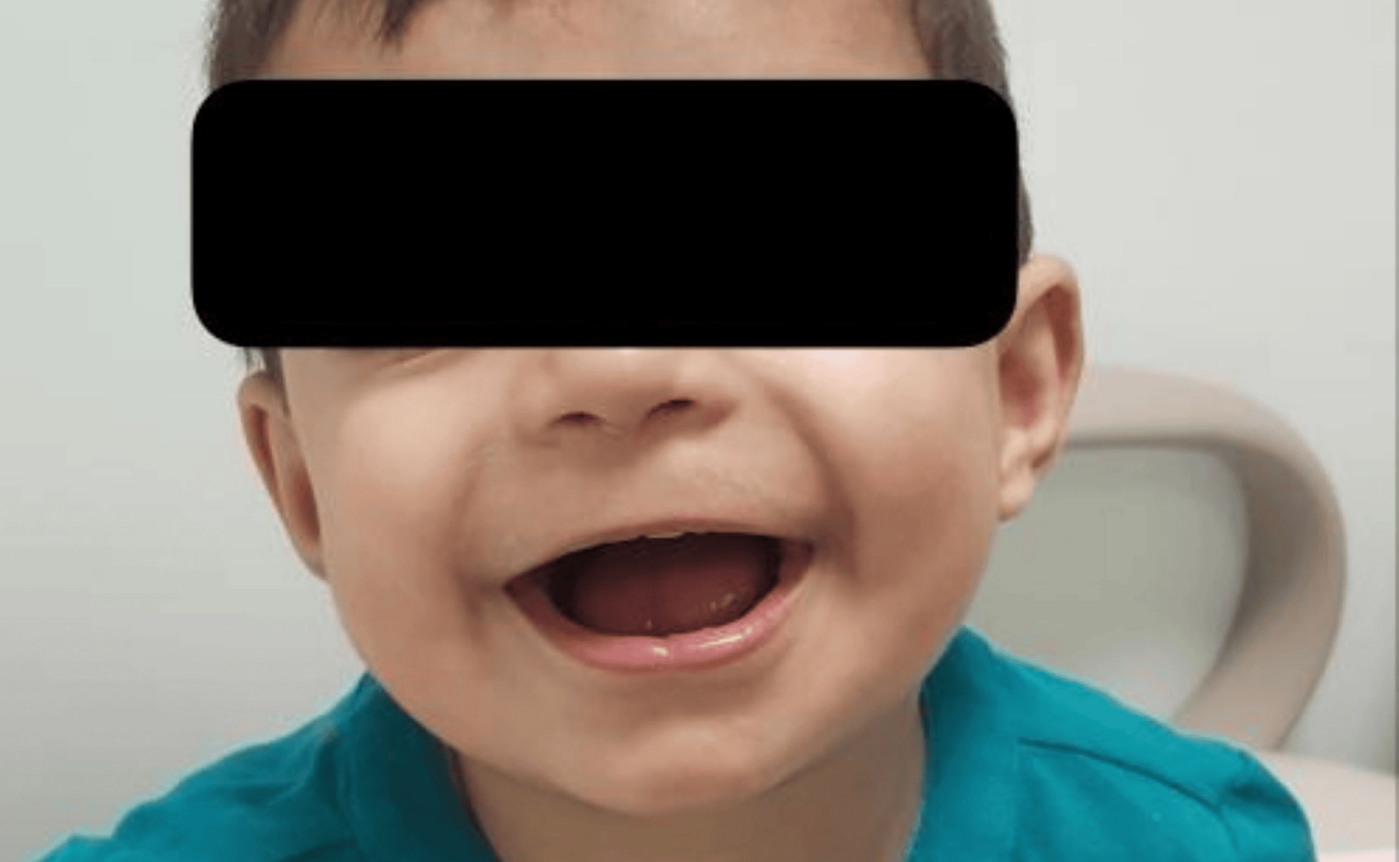
Seven-year-old Saudi boy with DeSanto-Shinawi Syndrome (case 2)

**Figure 3:**
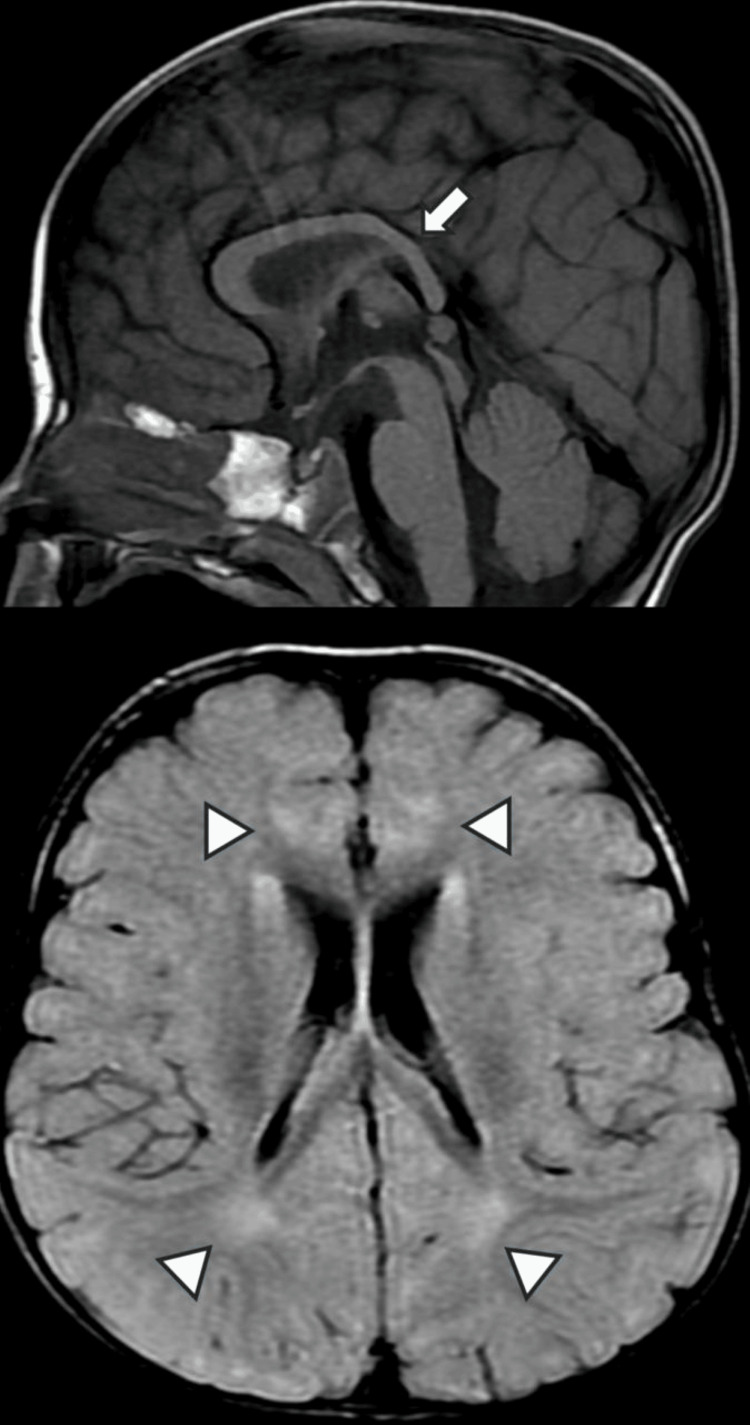
Brain MRI of case 2: Upper image of the midline sagittal T1 shows a thin corpus callosum, particularly the posterior body and splenium (white arrow). The lower axial flair image shows the reduced volume of white matter with abnormal hyperintensity of periventricular and deep white matter (white arrowheads)

This seven-year-old Saudi boy was born into a consanguineous marriage. He presented to the clinic in the first two years of life with intellectual, motor, and speech delay, sleep disturbances, and hypotonia (Table 1). Dysmorphic features included a square-shaped face, midface hypoplasia with bilateral ptosis, deep-set eyes, flat nasal bridge, bulbous nasal tip, and low-set ears (Figure 2). He had feeding difficulties constipation and eczema on the face and hands. Brain magnetic resonance imaging (MRI) showed an increased hyperintensity signal in periventricular (Figure 3). Whole-exome sequencing revealed a heterozygous novel variant in WAC gene c.921del (p. E307Dfs*24). Carrier testing of this variant for his parents showed a wild type.

Case 3

This five-year-old Omani boy born to a non-consanguineous marriage was a product of in vitro fertilization (IVF). His pregnancy and delivery were uneventful. He presented to the clinic in the first two years of life with intellectual, motor, and speech delays and had no hypotonia (Table 1). He had a history of recurrent seizures diagnosed as epilepsy upon showing an abnormal electroencephalogram. He showed dysmorphic features including a large broad forehead, square-shaped face, almond eyes, depressed nasal bridge, thick lips, and low-set ears. This patient had partial anomalous pulmonary venous return, unilateral left choanal atresia, flat feet, hypospadias, undescended testes, hydrocephalus with required insertion of ventriculoperitoneal (VP) shunt, and unilateral ectopic left kidney. Brain MRI showed subependymal cystic changes in the bilateral caudothalamic groove, left-sided periventricular leukomalacia, and delayed myelination.

Whole-exome sequencing was normal, but the array comparative genomic hybridization (ACGH) analysis revealed a 9,389 kbp deletion on chromosome 10p12.31p11.22 with 74 genes deleted including the WAC gene.

## Discussion

Since the first description of DESSH by DeSanto et al. in 2015, many publications across the globe replicated the genotypic and phenotypic characteristics of this disorder [1-9]. There has been only one case of DESSH reported from the Arab world [6]. Here, we present three patients from Arab countries, specifically Saudi Arabia and Oman.

In this report, we describe three cases with DESSHS, two of them had novel, de novo point mutation and the third had chromosomal deletion encompassing the WAC gene. They all exhibited phenotypes similar to that were described in previous reports including dysmorphic features, global developmental delay, and gastrointestinal symptoms [1-9]. Nevertheless, our patients showed other features that were not reported before with DESSHS. These include choanal atresia, flat feet, hypospadias, and MRI changes showing caudothalamic groove and periventricular leukomalacia. Further studies are needed to replicate our observation as some of these findings may be coincidental. For instance, premature infants with germinal matrix hemorrhage and other ischemic insults frequently have caudothalamic grooves and periventricular leukomalacia [13]. However, none of our patients was preterm or had perinatal asphyxia.

One of our patients presented with a limitation of the ankle joint movement since birth. A similar case was reported from Portugal in a patient with limited range of motion (ROM) since birth [14]. However, the Portuguese patient showed chronic polyarthritis ten years after DESSH was diagnosed whereas our patient did not show any signs of arthritis till his current age of eight. The presence of frequent respiratory and dermatological infections was described in previous reports [4,6]. We have not seen frequent infections in our patient; however, one of them (patient 2) had chronic eczema on his hands and feet, which was not clear whether it was related to DESSH or not.

Our genetic diagnostic strategy for these patients was to start with ACGH followed by WES if the diagnosis is not confirmed. ACGH confirmed a 9,389 kbp deletion on chromosome 10p12.31p11.22 with 74 genes deleted including the WAC gene in case 3. This microdeletion was reported before in patients with DESSHS [11]. ACGH was normal in the other two cases and WES revealed two-point variants in the WAC gene c.479 del C (p. Pro160fs*32) and c.921del (p. E307Dfs*24), which are both novel variants. Carrier testing for parents of the two patients showed that they carry the wild type confirming that these variants are de novo. 

Our patient with deletion at chromosome 10p12.31p11.22 had partial anomalous pulmonary venous return. Interestingly, a similar cardiac defect was reported in 2012 by Okamoto et al. before the first description of DESSHS in a patient with microdeletions at 10p11.23-p12.1 overlapped four genes, including WAC and phenotype similar to DESSHS with typical craniofacial abnormalities and global developmental delay [15]. Similarly, variable congenital heart disease was documented in previous case reports of DESSHS especially those with 10p12.1 microdeletion including the WAC gene [5]. 

Our patient with a deletion on chromosome 10p12.31p11.22 developed hydrocephalus required VP shunt like the case described by DeSanto et al. that showed bilateral prominence of the subarachnoid spaces consistent with benign external hydrocephalus and stable ventriculomegaly seen on brain MRI and had WAC loss-of-function mutations with 10p11.23 microdeletion [1]. 

Compared to the two patients with a point mutation, our patient with a deletion on chromosome 10p12.31p11.22 seems to have a more severe phenotype including hydrocephalus, partial anomalous pulmonary venous return, choanal atresia, and epilepsy. 

As shown by our three cases and other reports, it appears that the spectrum and manifestations of DESSH are highly variable with unclear genotype-phenotype correlation [1-9,11]. Exploring this further, Toledo-Gotor et al. compared the phenotypic features of patients with point mutations in the WAC gene versus a 10p12.1 microdeletion [11]. They concluded that malar flattening and prominent forehead are associated more with point mutation while facial dysmorphic features like synophrys, deep-set eyes, and depressed nasal bridge tend to be more common in WAC deletion [11]. We have not observed a clear difference in craniofacial dysmorphic features between our two patients with point mutation and the third one with 10p12.1 microdeletion.

## Conclusions

We report three cases with DESSHS presented with typical dysmorphic features, global developmental delay, and behavioral problems. Moreover, our patients showed other features not reported before with DESSHS such as choanal atresia, flat feet, hypospadias, caudothalamic groove, and periventricular leukomalacia. Furthermore, we detected two novel variants in our patients. This report contributes to further delineating the phenotypic and genotypic spectrum of this condition. More studies are needed to ascertain our observations.
